# Distal Kaplan Fiber Tenodesis Surgical Technique

**DOI:** 10.1016/j.eats.2023.07.004

**Published:** 2023-10-02

**Authors:** Mohammad Ayati Firoozabadi, Seyed Mohammad Milad Seyedtabaei, Pouya Tabatabaei Irani, Behzad Nejad Tabrizi, Mohammad Pourfarzaneh, B.E. Mohammadmahdi Ghasemian

**Affiliations:** Joint Reconstruction Research Center, Tehran University of Medical Science, Tehran, Iran

## Abstract

Anterior cruciate ligament tears are a common knee injury, and even after reconstruction, some patients may still experience instability in their knee. To address this, extra-articular reinforcement may be necessary to prevent anterior translation and internal rotation of the tibia. Kaplan fibers (KFs), which are the attachments of the iliotibial band to the distal femur, can help improve anterolateral rotatory stability in the knee, especially in greater degrees of knee flexion. Our technique for KF reconstruction involves making a 10-cm incision in the skin and subcutaneous tissue of the distal and lateral thigh. We release a 10-cm × 1-cm strip of the iliotibial band while maintaining its tibial insertion and then stitch the free end of the strip before attaching it to the distal femur using a ToggelLoc (Zimmer Biomet). The advantage of this technique is that the tension of the iliotibial band strip can be adjusted using the ToggelLoc, allowing the surgeon to increase tension in the graft as needed to achieve ideal tension in knee flexion.

Anterior cruciate ligament (ACL) tears are a common type of knee injury, and arthroscopic ACL reconstruction surgeries have become increasingly popular. However, even with advances in surgical techniques, up to 30% of patients still experience anterolateral rotatory knee instability after surgery.^1^ To address this, some surgeons have suggested performing anterolateral ligament (ALL) reconstruction or tenodesis in addition to ACL reconstruction,^2^ which has reduced the incidence of complaints about rotatory instability but still leaves some patients with this problem.

The anterolateral complex (ALC) of the knee includes not only the ALL but also the iliotibial band (ITB), which has been found to act as a restraint to anterior translation and internal rotation of the tibia. Kaplan fibers (KFs) are the attachments of the ITB to the distal femur and have been recognized as playing an important role in maintaining rotatory stability of the knee, particularly in greater degrees of knee flexion (around 30-90 degrees).^3^ There are 2 bundles of KFs, proximal KF (PKF) and distal KF (DKF), which originate from the ITB and are connected to the lateral distal femur at different distances from the lateral epicondyle. At the same time, the ALL is responsible for the anterolateral rotatory stability of the knee at 0 to 30 degrees of knee flexion.

According to Lutz et al.,^4^ the KFs hold the ITB against the lateral epicondyle, allowing the distal ITB to function like a ligament and tighten during internal tibial rotation. The DKF is located near the superior lateral genicular artery, making it a useful landmark for DKF tenodesis. However, it is important to note that the DKF is a nonisometric structure that is under tension at 90 degrees of flexion and relaxed during knee extension.

## Surgical Technique (With Video Illustration)

### Indication and Contraindications

DKF tenodesis is a surgical option for patients experiencing anterolateral rotatory knee instability after ACL reconstruction. However, before performing DKF tenodesis, it is important to confirm that the patient does not have any damage to the posterolateral corner (PLC) of the knee or lateral compartment cartilage, as these conditions are contraindications for the procedure. In the case of our patient, magnetic resonance imaging (MRI) revealed a tear in the KF.

**Fig. 1:**
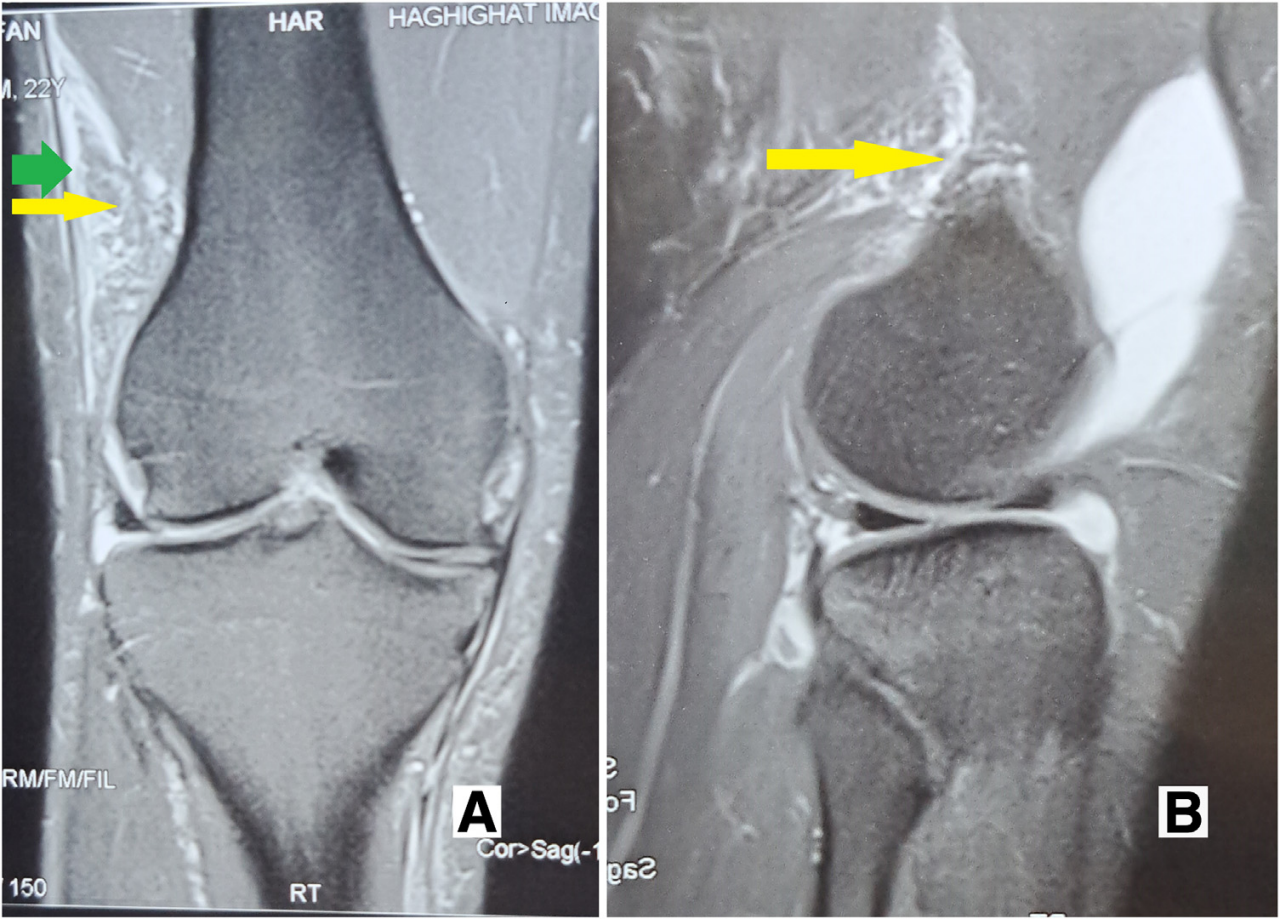
Coronal (A) and sagittal (B) complete tear of the Kaplan fiber complex (yellow arrow) indicated by the wavy appearance and surrounding edema green arrow = superficial iliotibial band (ITT).

To summarize, DKF tenodesis is a treatment option for patients with anterolateral rotatory knee instability after ACL reconstruction, but it is crucial to rule out any contraindications before proceeding with the surgery. In our patient’s case, a KF tear was identified on MRI (Fig 1).

### Procedure

The ACL reconstruction surgery is performed on the patient under spinal anesthesia in the supine position, with a tourniquet applied at an optimal pressure based on the patient’s systolic blood pressure (100 mm Hg above the patient’s systolic pressure). Depending on the patient’s condition, either a tibialis posterior allograft or hamstring autograft is used for the procedure, which is done using the transportal method. Graft fixation is performed in the distal femur and proximal tibia using Silva’s quadruple graft construct and suspensory button fixation technique with ToggleLoc and ToggleLoc XL (Zimmer Biomet).

**Fig. 2:**
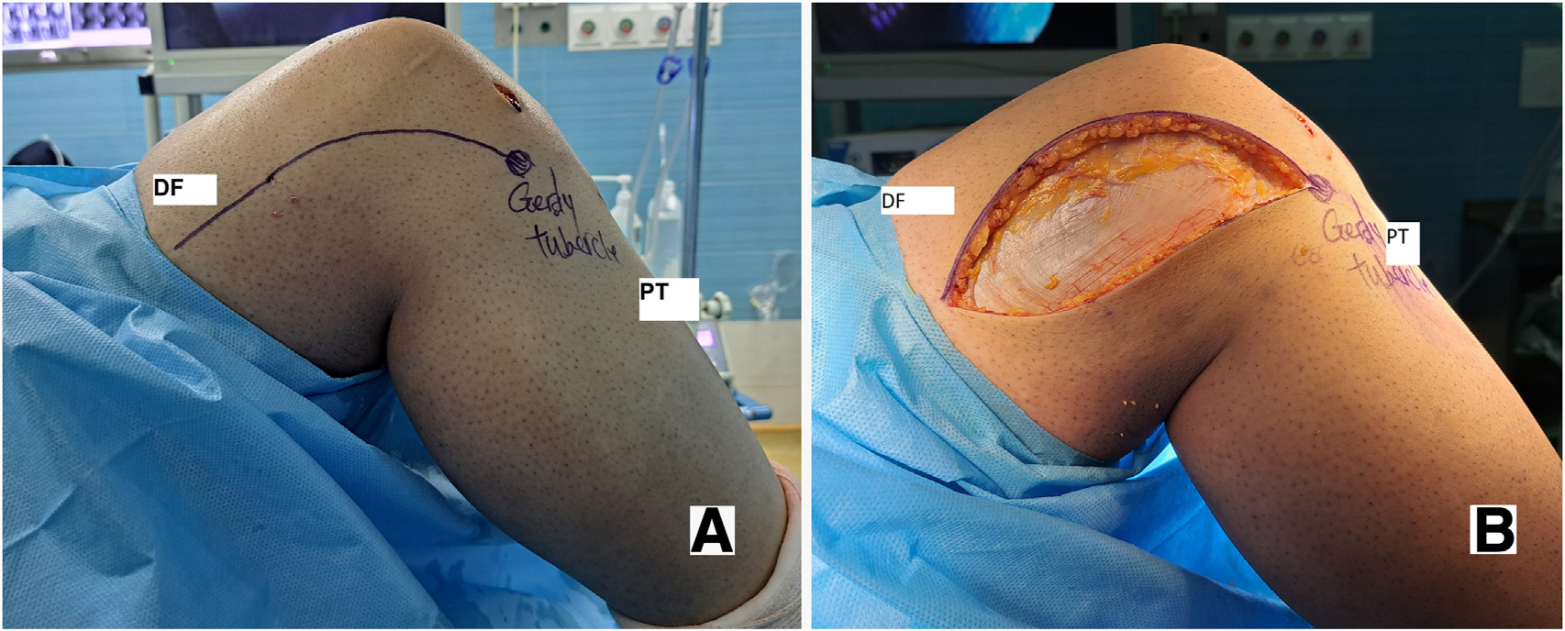
Skin marking before surgery (A) incision in lateral of the distal femur (B) (lateral view, right knee). (DF, distal femur; ITB, iliotibial band; PT, proximal tibia.)

**Fig. 3:**
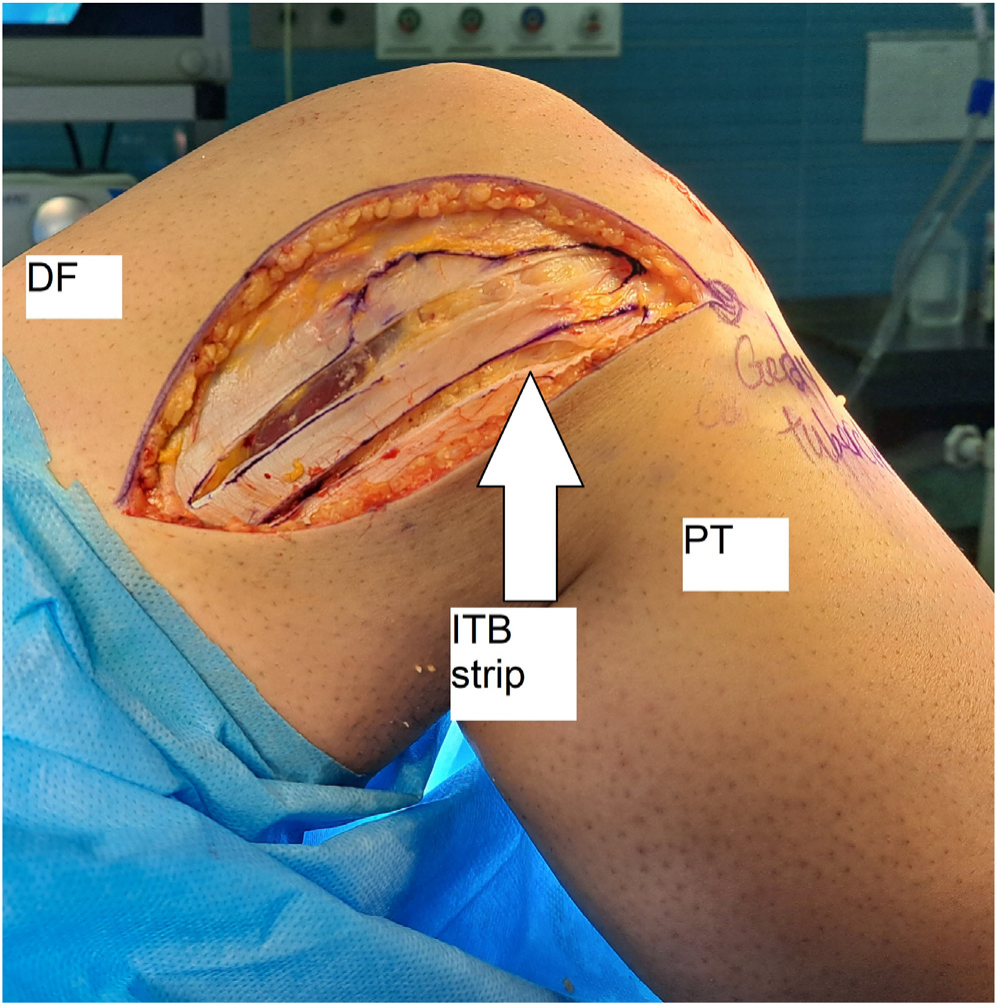
The 2 parallel incisions create a strip of iliotibial band of about 10 cm length for the graft (lateral view, right knee). (DF, distal femur; ITB, iliotibial band; PT, proximal tibia.)

After the ACL reconstruction surgery, an examination is performed to assess anterolateral rotatory stability. If the patient still experiences instability at more than 30 degrees of knee flexion and there are no contraindications, the patient may undergo DKF tenodesis surgery. This procedure involves making an incision of approximately 10 cm in length in the skin and subcutaneous tissues of the distal and lateral thigh, over the location of the middle portion of the ITB, and slightly curved toward the Gerdy tubercle (Fig 2). The skin flaps are then retracted, and the ITB’s posterior portion is identified through delicate dissection. Two incisions, each about 10 cm long, are made parallel to each other, with one 1 cm above the ITB’s posterior edge and the other 1 cm anterior to the first incision. The ITB must be attached to the distal femur (Fig 3).

**Fig. 4:**
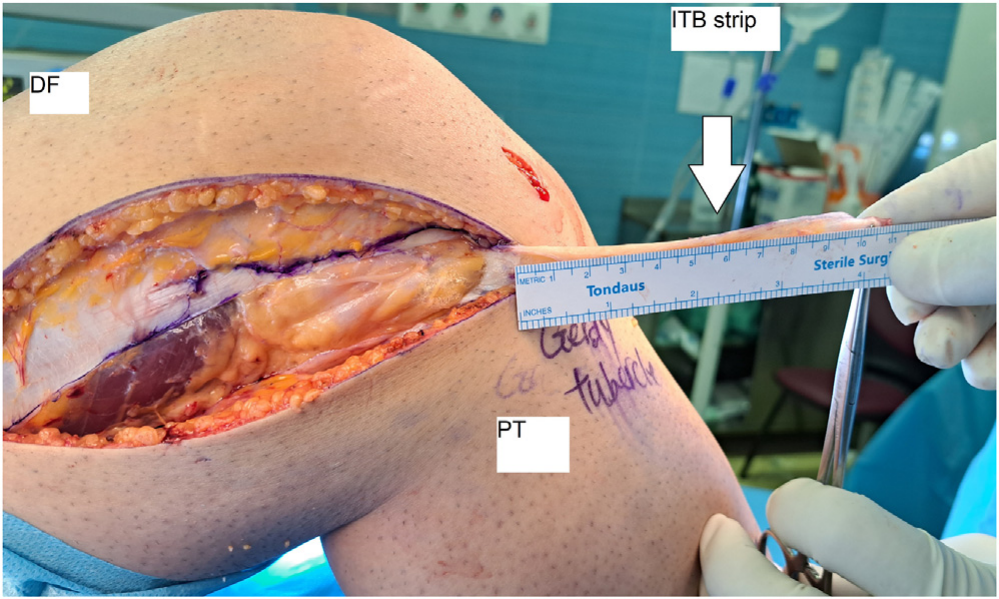
The 10-cm × 1-cm iliotibial band strip being dissected, keeping its tibial insertion at the Gerdy tubercle (lateral view, right knee). (DF, distal femur; ITB, iliotibial band; PT, proximal tibia.)

Next, a vertical incision is made at the most proximal region of the other cuts to release a 10-cm × 1-cm strip of the ITB while maintaining its tibial insertion at the Gerdy tubercle. This strip is then used for the DKF tenodesis surgery to help improve anterolateral rotatory stability in the knee (Fig 4).

**Fig. 5:**
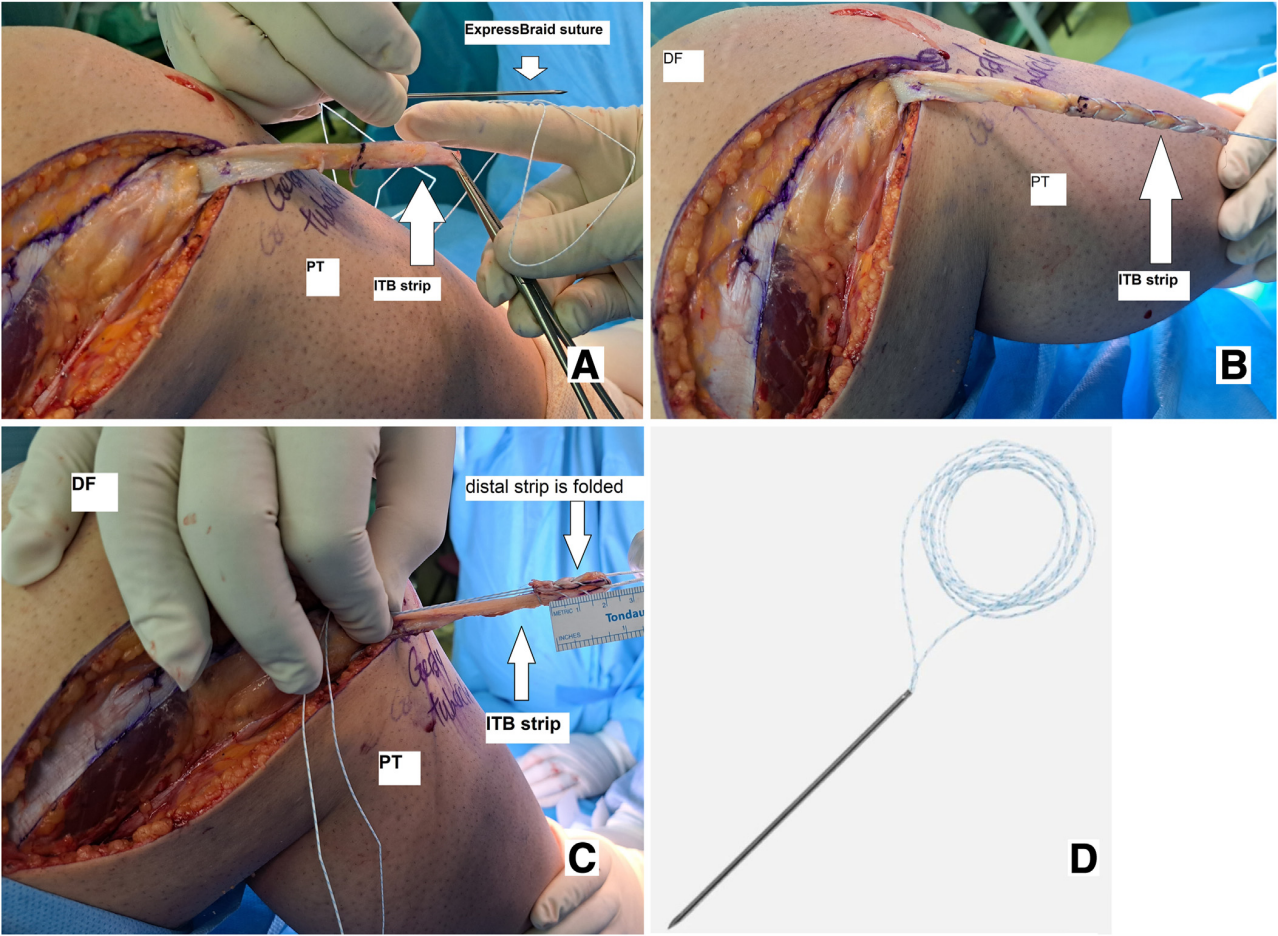
Stitching the free end of the strip of the iliotibial band over a length of 30 mm from distal to proximal, using a No. 2 ExpressBraid suture (A), stitched graft (B), folded distal strip (C), and ExpressBraid suture (D) (lateral view, right knee). (DF, distal femur; ITB, iliotibial band; PT, proximal tibia.)

**Fig. 6:**
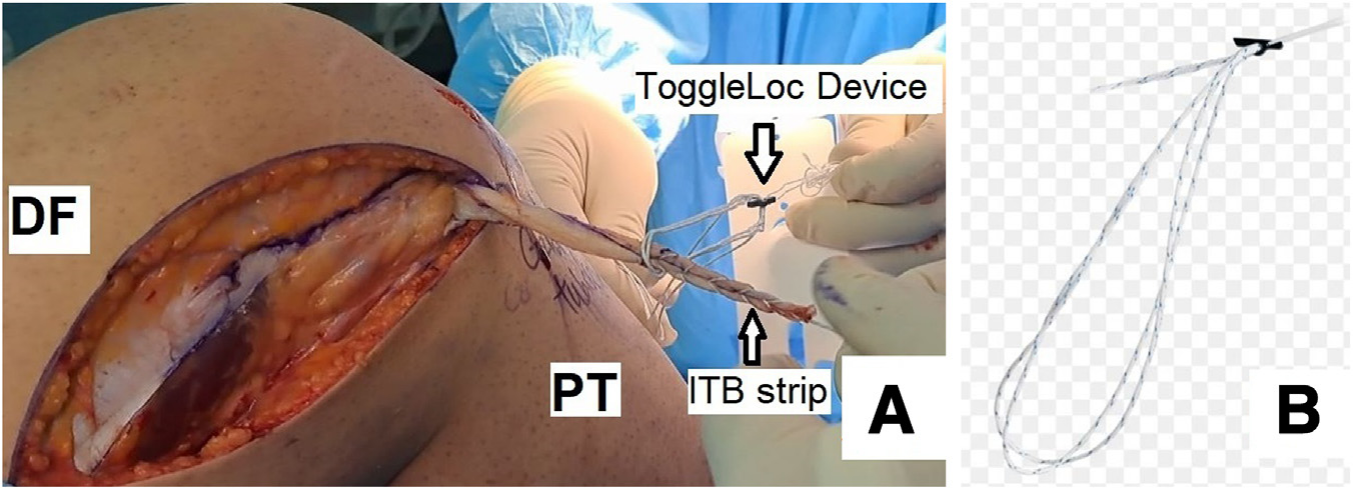
Passing the strip of the iliotibial band through the adjustable-length loop cortical button device (ToggleLoc device with ZipLoop technology) and suturing the 1.5-cm distal strip on the remaining 1.5 cm previously sutured with the help of the remaining No. 2 ExpressBraid (A) and adjustable-length loop cortical button device (B) (lateral view, right knee). (DF, distal femur; ITB, iliotibial band; PT, proximal tibia.)

To continue the procedure, the free end of the iliotibial band strip is stitched over a length of 5 cm from distal to proximal, using No. 2 ExpressBraid suture (Zimmer Biomet). Then, the strip is passed through an adjustable-length loop cortical button device, called a ToggleLoc device (Zimmer Biomet), and moved forward to 2.5 cm distally from the free end of the strip. A 2.5-cm distal strip is folded and sutured on the remaining 2.5 cm previously sutured using the remaining No. 2 ExpressBraid, ensuring that the ToggleLoc loop is accessible inside the sutured strip (Figs 5 and 6).^5^

**Fig. 7:**
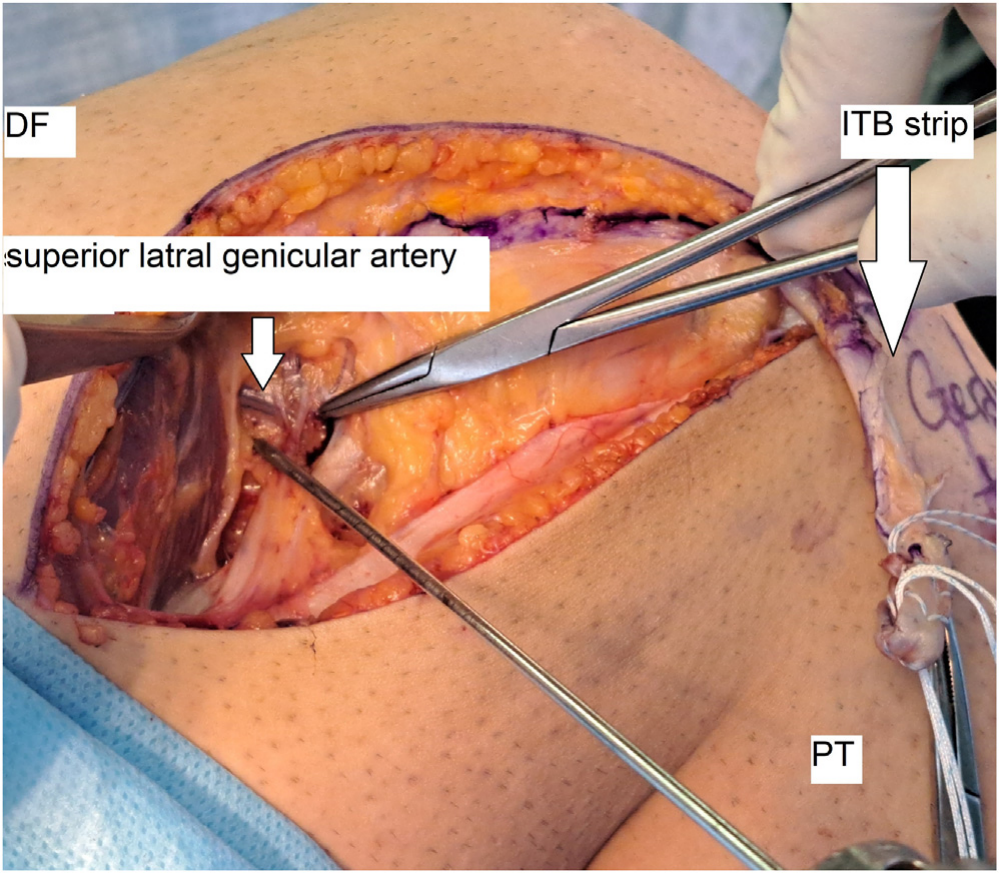
Location of insertion of the pin in the superior-to-superior lateral genicular artery (lateral view, right knee). (DF, distal femur; ITB, iliotibial band; PT, proximal tibia.)

Next, the distal part of the vastus lateralis muscle is bluntly defined and moved upward using a Bennett retractor to locate the superior lateral genicular artery below it. This artery is approximately 31 mm proximal to the lateral epicondyle. A 2.4-mm eyelet-tipped Kirschner wire is inserted with a 10-degree angle direction to the proximal and anterior sides at this point (Fig 7).

**Fig. 8:**
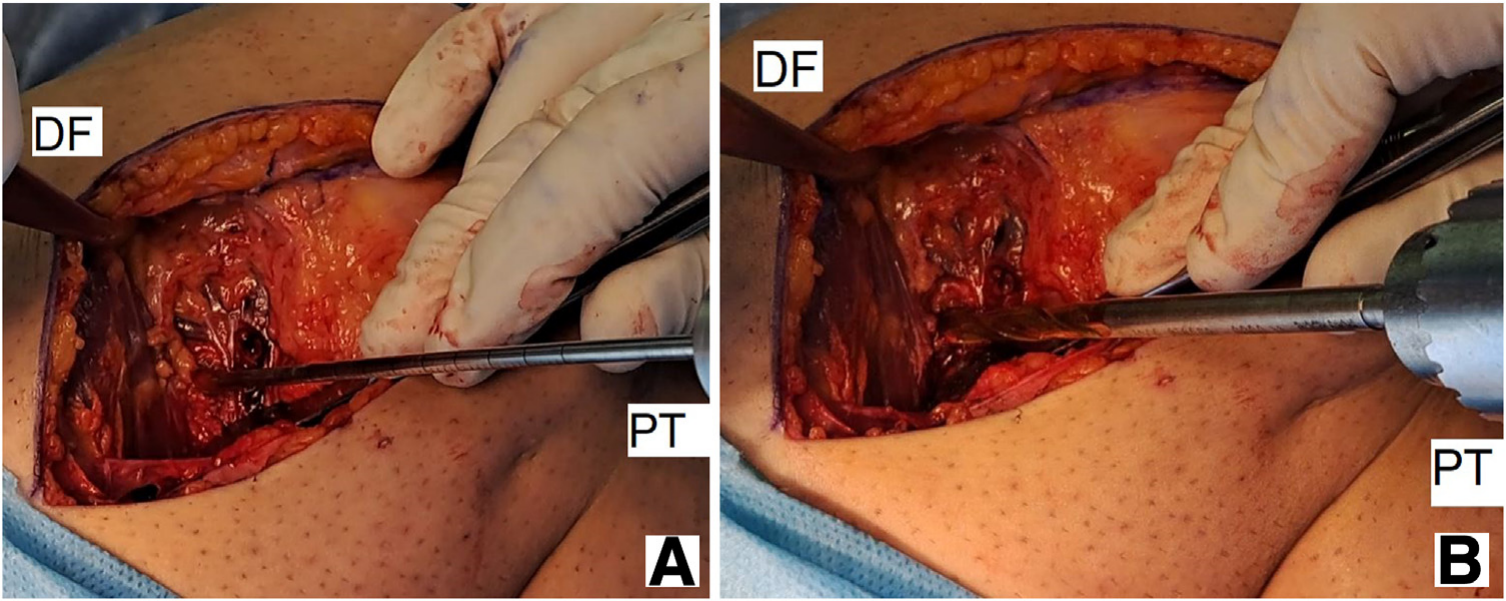
A cannulated drill is placed over the guide pin to ream the reconstruction graft tunnel (A) with reaming based on graft diameter (B) (lateral view, right knee). (DF, distal femur; ITB, iliotibial band; PT, proximal tibia.)

The entire pathway is then reamed using a 4.5 reamer on the pin, and the length of the strip from the eyelet-tipped Kirschner wire to the proximal side is determined by placing the prepared strip in tension near the femur bone. This length is usually between 1.5 and 2 cm. Using a sizer, the strip diameter that will be tied inside the bone tunnel is determined, and the reamer of the same size is used to ream over the eyelet-tipped Kirschner wire, equal to the length of the strip inside the tunnel (Fig 8).

**Fig. 9:**
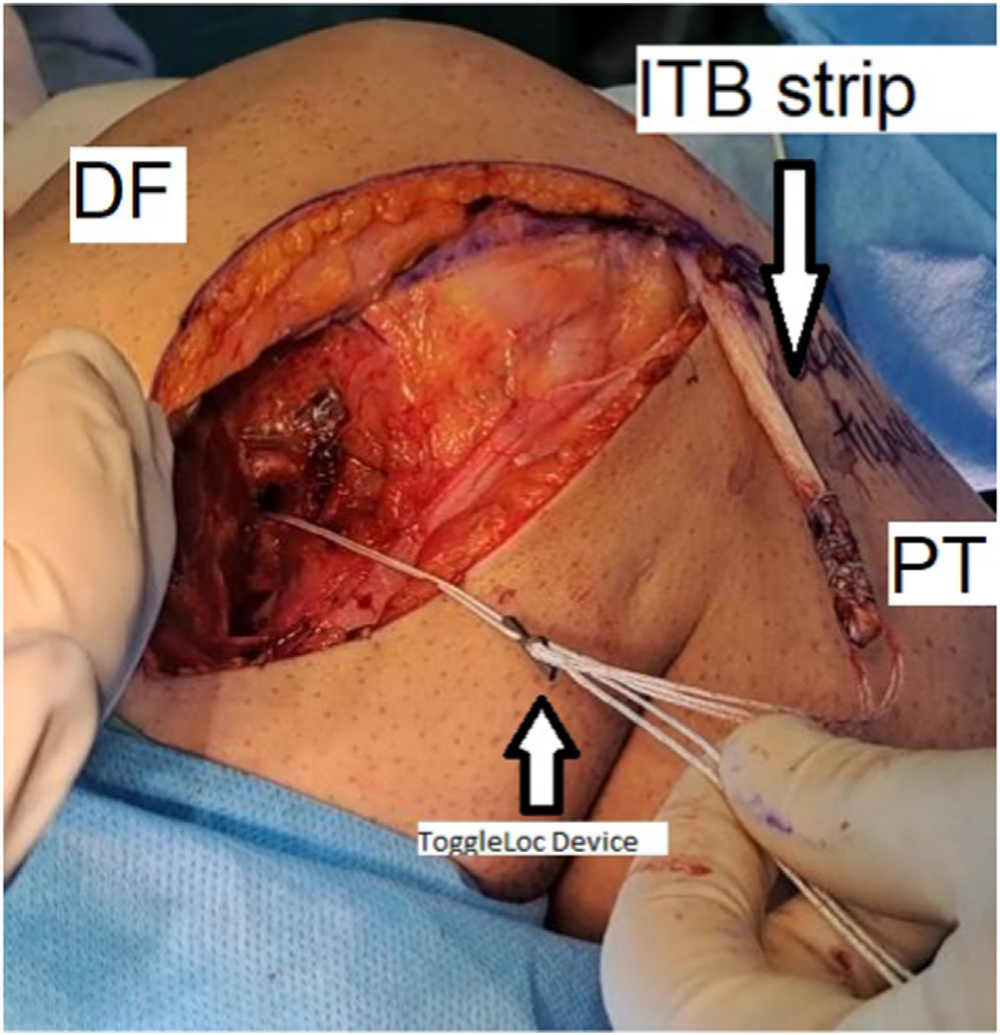
Fixation in the center of the Kaplan distal fiber insertion (lateral view, right knee). (DF, distal femur; ITB, iliotibial band; PT, proximal tibia.)

Then, using the eyelet-tipped Kirschner wire, the ToggleLoc, and the strip, strip passed through the tunnel that has been reamed. Before advancing the strip into the femoral tunnel, it is necessary to make sure that the cortical suspensory button is flipped. Once the strip is in place, the femoral pull sutures from the lateral side are tensioned to advance the strip up into the femoral tunnel (Fig 9).

**Fig. 10:**
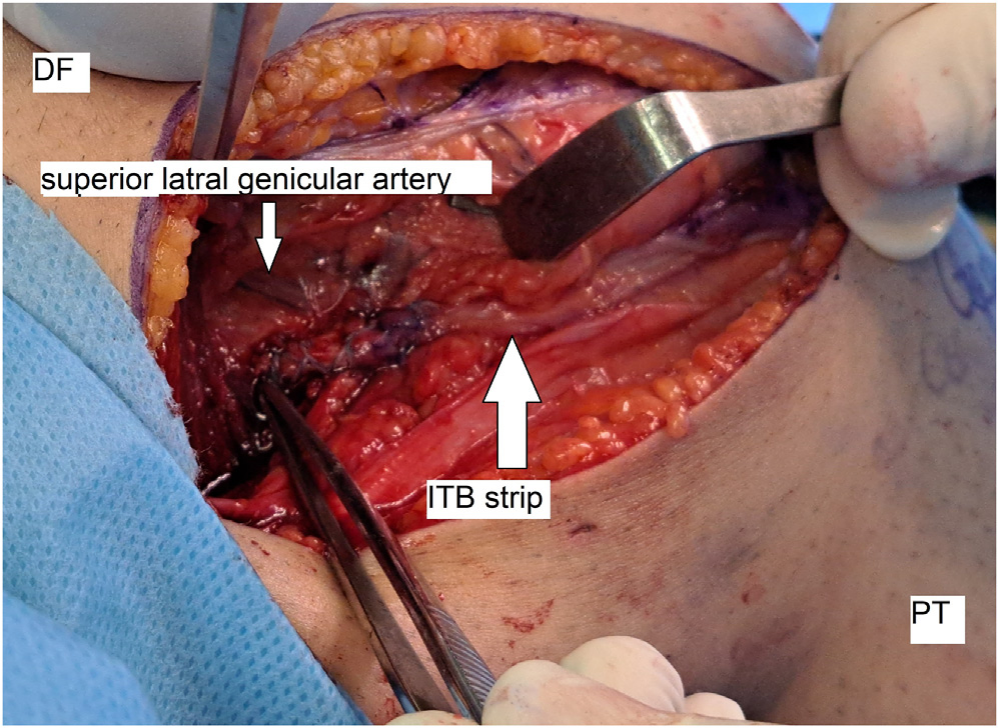
The end of the distal Kaplan fiber tenodesis (lateral view, right knee). (DF, distal femur; ITB, iliotibial band; PT, proximal tibia.)

It is important to note that during the final stage of the procedure, the knee should be kept in a 90-degree flexion position, and the foot should be in a neutral rotation position. Once the strip is in place, the knee is examined again for rotatory stability. If the conditions are acceptable, the tenodesis operation is concluded (Fig 10).

**Fig. 11:**
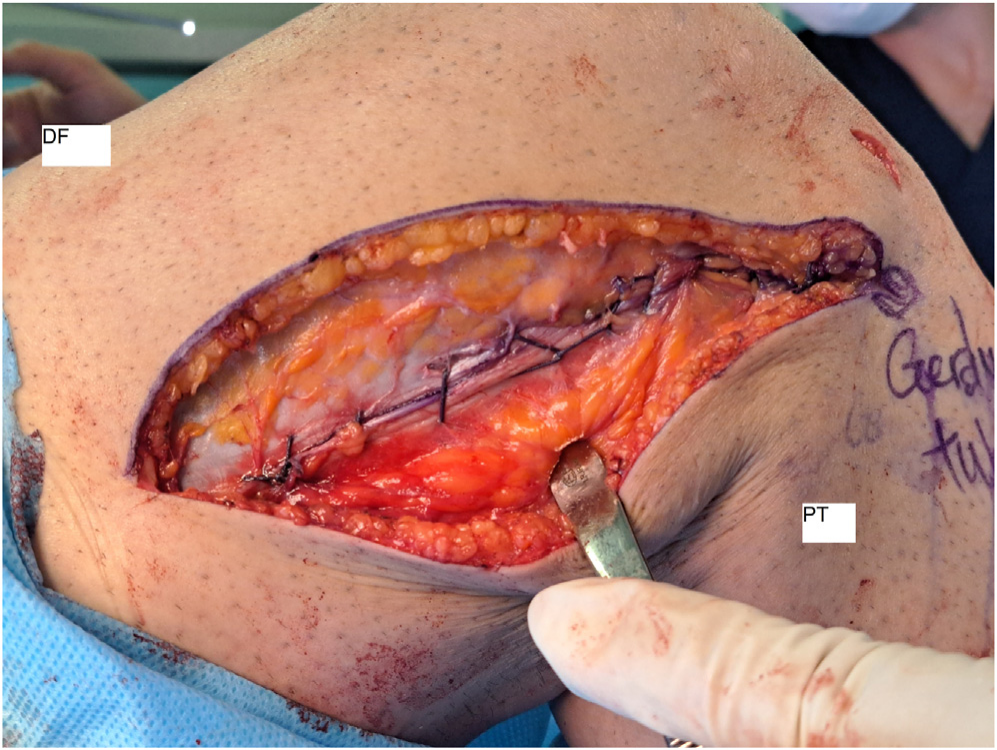
Iliotibial band graft defect closed (lateral view, right knee). (DF, distal femur; ITB, iliotibial band; PT, proximal tibia.)

**Fig. 12:**
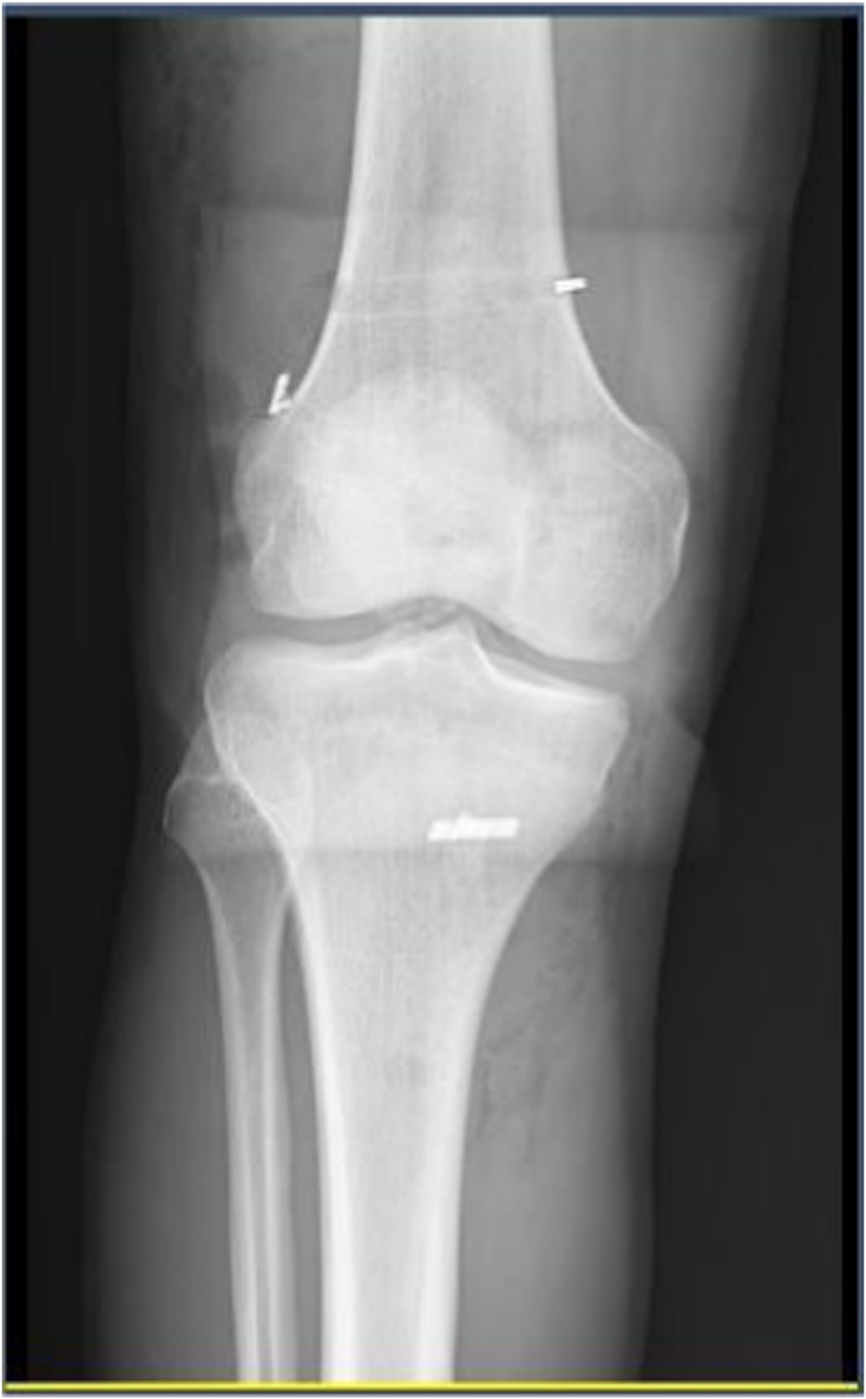
Postoperation knee radiography.

However, if the knee’s rotatory stability is still not acceptable with the benefit of this technique, the surgeon can increase the graft’s tension to 90 degrees of knee flexion to improve stability. It is worth noting that the graft is tight at 90 degrees of knee flexion but relaxed during knee extension. After the ITB defect is closed with Vicryl, the wound is closed with nylon and covered with a bandage (Fig 11). No drain is typically used, and postoperative knee radiography is performed to assess the procedure’s success (Fig 12). Table 1 lists the pearls and pitfalls of this technique, and a video (Video 1) is available for reference.

**Table 1:** Technique Pearls and Pitfalls

Pearls	Pitfalls
An incision of about 10 cm in length is made 1 cm above the ITB posterior edge	Difficult to dissect ITB and close ITB defect
The 10-cm × 1-cm ITB strip should be carefully released until its tibial insertion	Cutting strip from distal
The distal part of the vastus lateralis is defined bluntly and to identify the superior lateral genicular and reaming the entire path is with a 4.5 reamer	Superior lateral genicular artery injury creates bleeding and hematoma
The strip length that passes from the eyelet-tipped Kirschner wire to the proximal side is determined by placing the prepared strip in tension near the femur bone. This amount is the part that will be placed inside the bone tunnel	Miscalculating graft length incorporated in the femoral bone could be too short or too long
The ITB strip should be fixed to the femur with the knee at 90 degrees of flexion and neutral rotation	Joint over constrained, lateral patellar tilt

ITB, iliotibial band.

### Postoperative care

After the surgery, the patient is discharged the day after, and it is recommended to begin weightbearing immediately. The patient is referred to physical therapy, which follows the same regimen as the one performed for isolated ACL reconstruction.

## Discussion

The main finding of this technique is that DKF tenodesis with ITB can be used to improve extra-articular knee anterolateral rotatory stability. One of the advantages of this technique is that the tension of the ITB strip is adjustable using the ToggleLoc device (Zimmer Biomet), which allows the surgeon to increase the tension of the graft to achieve ideal tension in knee flexion. When fixing the strip, the knee should be at 90 degrees, which is the best degree for illiotibial band graft fixation and in a neutral position.^6^ Limited changes in lateral patellar tilt and patellofemoral contact pressures have been verified at this degree.^7^ Once the strip is fixed, it relaxes during external leg rotation and is tensioned during internal rotation, which helps prevent anterolateral rotation instability. Fixation with the ToggleLoc device (Zimmer Biomet) also allows for early mobilization to avoid joint stiffness.

However, this technique has some limitations. First, it requires an additional skin incision to perform the KF tenodesis. Second, there may be a confluence between the tunnels used to fix the ITB strip and the tunnels used for ACL reconstruction. Third, this technique may cause a small increase in pain, a slight reduction in quadriceps strength, and diminished subjective functional recovery for up to 1 to 2 months after the surgery.^8^

Table 2 compares the advantages and disadvantages of using the ToggleLoc device (Zimmer Biomet) vs an interference screw for KF tenodesis.

**Table 2:** Kaplan Fiber Tenodesis With ToggleLoc Device Advantages and Interference Screw Disadvantages

ToggleLoc Device Advantages	Interference Screw Disadvantages
Adjustable tension graft	Nonadjustable tension graft
After the graft is fixed, it is possible to add tension to the graft	After the interference screw is fixed, it is not possible to add tension to the graft
Fixation is more stable	Fixation may not completely stable because of metaphyseal bone fixation
Less reaming diameter	More reaming diameter
No soft tissue reaction	Soft tissue reaction by using the interference screw
Less fracture due to the lower diameter of the tunnel	More fracture due to the increased diameter of the tunnel

## Supplementary Data

ICMJE author disclosure forms

Video 1 Our technique for distal Kaplan fiber tenodesis. An incision of about 10 cm in length is made in the distal and lateral thigh over the location of the middle portion of the iliotibial band (ITB). An incision of about 10 cm in length is made 1 cm above the ITB posterior edge. Another similar parallel incision is performed on the ITB, 1 cm anterior to the first one. Then, a vertical incision on the most proximal region of the other cuts is performed to release a 10-cm × 1-cm ITB strip, maintaining its tibial insertion. The free end of the strip of the ITB is stitched over a length of 5 cm from distal to proximal, using the No. 2 ExpressBraid suture. The strip of the iliotibial band is passed through the adjustable-length loop cortical button device. Then, a 2.5-cm distal strip is folded and sutured. A 2.4-mm eyelet-tipped Kirschner wire is inserted with a 10-degree angle direction to the proximal and anterior sides of the lateral geniculate artery. Then, the entire path is reamed with a 4.5 reamer and then reamed equal to the length of the strip inside the tunnel. The ToggelLoc and the strip are passed through the tunnel, and the femoral pull sutures are tensioned from the lateral side to advance the strip up into the femoral tunnel. The graft in 90 degrees of knee flexion is tight and relaxed in the extension of the knee.
